# Risk analysis of gravity dam instability using credibility theory Monte Carlo simulation model

**DOI:** 10.1186/s40064-016-2508-7

**Published:** 2016-06-18

**Authors:** Cao Xin, Gu Chongshi

**Affiliations:** State Key Laboratory of Hydrology and Water Resources and Hydraulic Engineering, Hohai University, Nanjing, 210098 Jiangsu People’s Republic of China; National Engineering Research Center of Water Resources Efficient Utilization and Engineering Safety, Hohai University, Nanjing, 210098 Jiangsu People’s Republic of China; College of Water Conservancy and Hydropower Engineering, Hohai University, Nanjing, 210098 Jiangsu People’s Republic of China

**Keywords:** Gravity dam, Anti-sliding stability, Risk analysis, Fuzzy sets, Credibility theory

## Abstract

Risk analysis of gravity dam stability involves complicated uncertainty in many design parameters and measured data. Stability failure risk ratio described jointly by probability and possibility has deficiency in characterization of influence of fuzzy factors and representation of the likelihood of risk occurrence in practical engineering. In this article, credibility theory is applied into stability failure risk analysis of gravity dam. Stability of gravity dam is viewed as a hybrid event considering both fuzziness and randomness of failure criterion, design parameters and measured data. Credibility distribution function is conducted as a novel way to represent uncertainty of influence factors of gravity dam stability. And combining with Monte Carlo simulation, corresponding calculation method and procedure are proposed. Based on a dam section, a detailed application of the modeling approach on risk calculation of both dam foundation and double sliding surfaces is provided. The results show that, the present method is feasible to be applied on analysis of stability failure risk for gravity dams. The risk assessment obtained can reflect influence of both sorts of uncertainty, and is suitable as an index value.

## Background

Risk analysis of gravity dam anti-sliding instability involves factors of complicated uncertainty. In traditional analysis method, main influencing factors of gravity dam instability (e.g. various imposed loads, material properties and geometric parameters) are all viewed as random variables (Peyras et al. 2012). Now researchers gradually realize that fuzziness is significant in risk analysis of gravity dam instability, including: fuzzy stability failure limit state (Su et al. 2009), fuzzy mechanical parameters of dam foundation (Hua and Jian 2003), fuzzy safety monitoring data of dam and dam foundation (de la Canal and Ferraris 2013; Gu et al. 2011) etc. Therefore, problems of calculating precise failure risk ratio of gravity dam instability influenced by complicated uncertainties and determining corresponding indexes and standards need to be solved.

Since gravity dam involves factors of complicated uncertainty during operation, researchers have applied possibility theory (Zadeh 1965, 1996) into stability failure risk analysis of gravity dam (Su and Wen 2013; Haghighi and Ayati 2016; Li et al. 2011; Sadeghi et al. 2010). Corresponding models have been established to consider both randomness and fuzziness of influence factors. However, in current studies, fuzziness and randomness of factors influencing gravity dam stability are studied separately, which makes the gained risk assessments are described jointly by probability measure and possibility measure. For example, a certain failure risk probability interval is $[\tilde{R}_{L} ,\tilde{R}_{U} ]$ with possibility $\alpha = 0.5.$ Given that probability and possibility are independent, this description fails to synthetically reflect the contribution of fuzzy factors to risk assessments, and is difficult to clearly characterize the likelihood of risk occurrence in practical engineering.

Actually, fuzziness and randomness of influence factors have no essential difference in stability failure risk analysis of gravity dam. They shall be processed in the same frame. Therefore, this article views gravity dam anti-sliding stability failure as a hybrid event, uses credibility theory (Liu 2006) to consider both randomness and fuzziness of failure criterion, design parameters and measured data simultaneously and establishes credibility stability failure risk analysis model of gravity dam to objectively reflect both sorts of uncertainty. Combining present analysis model with Monte Carlo simulation, calculation method and procedure are proposed to analyze the risk of anti-sliding stability of gravity dam.

## Instability credibility risk ratio of gravity dam

Instability risk calculation of gravity dam based on Monte Carlo simulation can be divided into four steps: (1) determine the instability model function of gravity dam; (2) identify and quantify uncertainty of model factors; (3) simulate; (4) analyze results and calculate instability risk ratio.

### Instability risk calculation mode of gravity dam

Gravity dam instability is regarded as a random fuzzy event. Fuzziness in the calculation model comes from model factors and failure criterion. There will be three conditions: failure criterion is determined while the model contains both random and fuzzy factors; failure criterion is fuzzy and the model contains random factors only; failure criterion is fuzzy and the model contains both random and fuzzy factors.

Stability state function of gravity dam is expressed by *Z* and fuzzy failure criterion is expressed by credibility density distribution function $\eta \left( Z \right).$ Then, credibility risk ratio model considering both fuzzy failure criterion and fuzzy model factors is:

1 $$\tilde{P} = \mathop \int \nolimits_{ - \infty }^{ + \infty } \eta \left( Z \right)\varphi \left( Z \right)dZ$$

where $\varphi \left( Z \right)$ is credibility density distribution function of state function and $\eta \left( Z \right)$ is credibility failure criterion. Through appropriate conversion, failure probability model under rest two conditions can be gained as following:

1. State function is random fuzzy variable and failure criterion is expressed by the limit state function. In other words, when $Z < K,$ the structure failed and the failure criterion is $$\eta \left( Z \right) = \left\{ {\begin{array}{*{20}l} {1,} \hfill & {{\text{if}}\, Z < K} \hfill \\ {0,} \hfill & { {\text{if}}\, Z \ge K} \hfill \\ \end{array} } \right.$$. Then, the credibility risk ratio is degraded into:
  2 $$\tilde{P}(Z < K) = \mathop \int \nolimits_{ - \infty }^{K} \varphi \left( Z \right)dZ = \varPhi \left( K \right).$$

The state function contains random variables only and the failure criterion is fuzzy. According to the integrated chance theorem ${\text{Ch}}\left\{ {\varTheta \times Y} \right\}{\text{ = Pr}}\{ Y\} ,$ the credibility risk ratio degraded into:

3 $$\tilde{P} = \mathop \int \nolimits_{ - \infty }^{ + \infty } \eta \left( Z \right)f\left( Z \right)dZ$$

where $f\left( Z \right)$ is random distribution density function of the state function. Failure risk ratio is a real number expressed by credibility distribution.

Since credibility failure criterion distribution function is often sectionally derivable and derived functions are sectionally continuous, it can get from partial integration of Eq. (1):

4 $$\tilde{P} = \mathop \int \nolimits_{a}^{b} \varPhi \left( Z \right)\left( { - \dot{\eta }\left( Z \right)} \right)dZ$$

where *a* and *b* are lower and upper limits of fuzzy interval of failure criterion. Derived function of $\eta \left( Z \right)$ is continuous in the interval $\left[ {a,b} \right].$ When $Z < a,\, \eta \left( Z \right) = 1.$ When $Z > a,\, \eta \left( Z \right) = 0.$

### Instability model function of gravity dam

Functional status of structure generally can be expressed by performance function:

5 $$Z = g\left( {X_{1} ,X_{2} , \ldots ,X_{n} } \right)$$

where $X_{i} \left( {i = 1,2, \ldots ,n} \right)$ is actions that could influence the structure and environmental influence as well as performance and geometric parameters of materials and rock soils.

For the simplest situation, Eq. (5) can be rewritten into:

6 $$Z = R /S$$

where *R* is resistance of structure and *S* is load effect of structure.

**Fig. 1:**
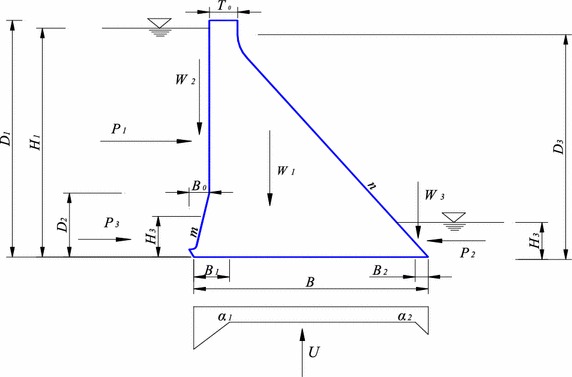
Anti-sliding stability analysis of gravity dam foundation

Main variables of instability risk ratio of gravity dam foundation include upstream and downstream water levels $H_{1}$ and $H_{2}$, silt elevation $H_{3}$, uplift pressure reduction coefficient $\alpha_{1}$ and $\alpha_{2} ,$ shearing friction coefficient $f^{{\prime }}$ and cohesion $c^{{\prime }}$ of the contact surface between dam concrete and dam foundation, volume weight of concrete $\gamma_{c}$ and dam profile size, and the force condition is shown in Fig. 1. Shearing strength state function is:

7 $$Z = R /S = \left( {f^{'} \sum W + c^{{\prime }} A} \right) /\sum P = g\left( {H_{1} ,H_{2} ,H_{3} ,\alpha_{1} ,\alpha_{2} ,f^{{\prime }} ,c^{{\prime }} ,\gamma_{c} ,A} \right)$$

In Fig. 1, $W_{1} ,\,W_{2} ,\,W_{3}$ are upstream vertical water pressure, dead weight of dam block and downstream vertical water pressure respectively. $P_{1} ,\,P_{2}$ and $P_{3}$ are respectively upstream horizontal water pressure, downstream horizontal water pressure and horizontal silt pressure. $U$ is uplift pressure. The calculation expressions of these forces can be derived as follows:

$$\begin{aligned} & \sum W = W_{1} + W_{2} + W_{3} = \gamma_{c} \left[ {\frac{1}{2}D_{1} \left( {B - B_{0} } \right) + \frac{1}{2}mB_{0}^{2} + \frac{1}{2}nT_{0}^{2} } \right] + \gamma_{w} \left( {H_{1} B_{0} - \frac{1}{2}mB_{0}^{2} } \right) + \frac{1}{2}\gamma_{w} nH_{2}^{2} \\ & \sum P = P_{1} - P_{2} + P_{3} = \left[ {\left( {H_{1}^{2} - H_{2}^{2} } \right)\gamma_{w} } \right] /2 + H_{3}^{2} \gamma_{s} /2 \\ & U = \frac{1}{2}\gamma_{w} \left( {H_{1} B_{1} + \alpha_{1} H_{1} B + \alpha_{2} H_{2} B + H_{2} B_{2} } \right) \\ \end{aligned}$$

where $\gamma_{c} ,\,\gamma_{w}$ and $\gamma_{s}$ are unit weight of concrete, water and silt respectively.

**Fig. 2:**
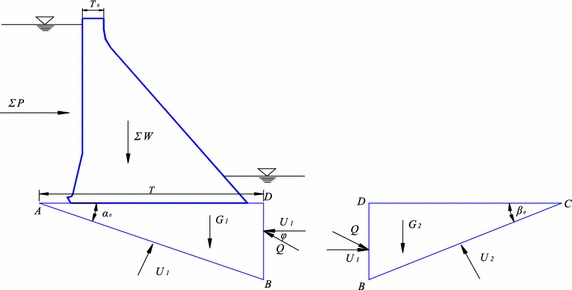
Anti-sliding stability analysis of double sliding surfaces of gravity dam

During deep anti-sliding stability analysis of both surfaces of gravity dam, a deep weak structural surface AB is chosen (Fig. 2), which is called the main slip surface. BC is an assistant plane of fracture. Stresses on ABD are dam weight $(W),$ total horizontal load of dam (*H*), dead load of block ABC $\left( {G_{1} } \right)$, uplift pressure of AB $\left( {U_{1} } \right)$, seepage pressure on the ABC–BCD interface $\left( {U_{3} } \right)$, acting force between sliding blocks (*Q*). Stresses in BCD include opposite acting force (*Q*), seepage pressure $\left( {U_{3} } \right)$, dead load of block BCD $\left( {G_{2} } \right)$, and uplift pressure of BC $\left( {U_{2} } \right)$.

Shearing strength of ABD is:

8 $$Z_{1} = \frac{{f_{1}^{'} \left[ {\left( {W + G_{1} } \right)cos\alpha_{0} - \sum Psin\left( {\varphi - \alpha_{0} } \right) - Qin\left( {\varphi - \alpha_{0} } \right) - U_{1} + U_{3} sin\alpha_{0} } \right] + c_{1}^{'} A_{1} }}{{\left( {W + G_{1} } \right)sin\alpha_{0} + \sum Pcos\alpha_{0} - U_{3} cos\alpha_{0} - Qcos\left( {\varphi - \alpha_{0} } \right)}}$$

Shearing strength of BCD is:

9 $$Z_{2} = \frac{{f_{2}^{'} \left[ {G_{2} { \cos }\beta_{0} + Q{ \sin }\left( {\varphi + \beta_{0} } \right) - U_{2} + U_{3} { \sin }\alpha } \right] + c_{2}^{'} A_{2} }}{{Q{ \cos }\left( {\varphi + \beta_{0} } \right) - G_{2} { \sin }\beta_{0} + U_{3} { \cos }\beta_{0} }}$$

where $A_{1}$ is area of structural surface AB and $A_{2}$ is area of assistant plane of fracture BC. The calculation expressions can be derived as follows:

$$\begin{aligned} A_{1} & = bT / {\text{cos}}\alpha_{0} \\ A_{2} & = bT{ \tan }\alpha_{0} / {\text{sin}}\beta_{0} \\ \end{aligned}$$

For the loading condition shown in Fig. 2, $\sum W$ and $\sum P$ are the same as previously mentioned. The calculation expressions of the rest can be derived as:

$$\begin{aligned} G_{1} & = \gamma_{s} bT^{2} { \tan }\alpha /2 \\ G_{2} & = \gamma_{s} b{ \cot }\beta (T{ \tan }\alpha )^{2} /2 \\ \end{aligned}$$

where *b* is width of dam block.

According to equal safety coefficient method, when ABC and BCD reach the limit state at the same time, the double sliding surface model will surface buckling failure. In other words, the limit state function meets $Z = Z_{1} = Z_{2} .$ Based on this implicit function, thrust *Q* and the limit state function value *Z* can be calculated.

### Risk identification of model factors

Uncertainty of model factors has to be analyzed when discussing instability process of gravity dam using the opinion of mixed uncertainty. Gravity dam, a complicated structural system, will suffer various concentrated forces and distributed forces (e.g. dead load, hydraulic pressure and seepage pressure of dam foundation) during construction and operation. Man causes of gravity dam instability include flood, earthquakes, seepage of dam foundation, material aging, and so on. The following text analyzes uncertainty of water level and material parameters.

Water level is a random variable. Upstream water level is related with pondage, reservoir inflow and discharge flow, while downstream water level is mainly influenced by discharge flow. Reservoir inflow is affected by rainfall and shows high uncertainty. Discharge flow is controlled by hydraulic parameters and also shows certain uncertainty. Additionally, hydrometry has system and observation errors, which shows some uncertainty. Dam water level during the service period is estimated through statistical analysis of complete monitoring sequences, which could use the classic frequency approach to process it uncertainty. Upstream and downstream water levels can be viewed as random variables (Salmon and Hartford 1995) which obey a certain probability distribution.

Material parameters involved in the instability risk analysis of gravity dam include volume weights of dam and rocks as well as shearing friction coefficient and cohesion of dam foundation and slip surface. Volume weight of dam concrete was determined by test. Sample size met requirement of statistical analysis and was treated as a random variable. Uncertainty of mechanical parameters of rocks in dam foundation includes: (1) uncertainty of engineering geological investigation; (2) uncertainty of engineering geological rock group and rock structure. Fuzziness of mechanical parameters of rocks, statistical variables that combines survey crew experiences and rock grouping, has more important significance.

### Uncertainty of failure criterion

Buckling failure of gravity dam is a progressive force when the yield range expands from local to global. Some artificial influences exist in understanding the failure criterion (Ma and Wu 2001). According to limit equilibrium state method, failure state of sliding surface is divided clearly by the limit state function $Z = K.$ When safety coefficient of the sliding surface reaches a specific value $K,$ the whole system reaches the ultimate equilibrium state. However, fuzzy number is more practical to express the progressive process. When considering fuzzy failure criterion, fuzzy limit state of the structure is possibility distribution that obeys to a certain membership function. For example, the membership distribution function $\mu \left( Z \right)$ is a symmetric triangular distribution:

10 $$\mu \left( Z \right) = \left\{ {\begin{array}{*{20}l} {\frac{{2\left( {Z - a} \right)}}{b - a}, } \hfill & \quad {{\text{for}}\; a \le Z \le \frac{a + b}{2}} \hfill \\ {\frac{{2\left( {b - Z} \right)}}{b - a}, } \hfill & \quad {{\text{for }}\; \frac{a + b}{2} \le Z \le b} \hfill \\ {0 ,} \hfill & \quad {{\text{for}} \;{\text{otherwise}}} \hfill \\ \end{array} } \right.$$

According to definitions of credibility measure and credibility distribution, credibility distribution density function corresponding to the possibility distribution of failure criterion is:

11 $$\eta \left( Z \right) = \left\{ {\begin{array}{*{20}l} {1,} \hfill & \quad {{\text{for}} \;Z \le a} \hfill \\ {\frac{Z - a}{b - a},} \hfill & \quad {{\text{for}}\; a < Z \le b} \hfill \\ {0,} \hfill & \quad {{\text{for}}\; Z > b} \hfill \\ \end{array} } \right.$$

**Fig. 3:**
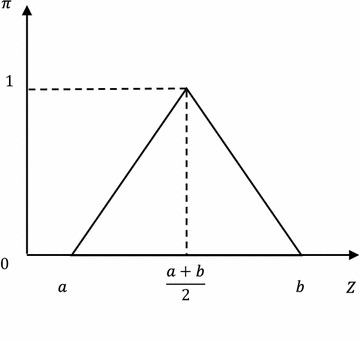
Distribution of fuzzy failure criterion

**Fig. 4:**
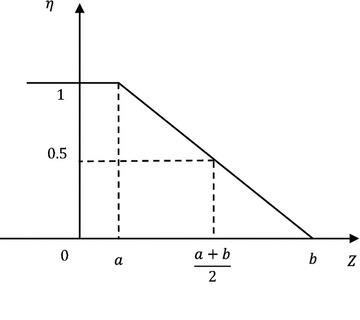
Lower semi-trapezoid distributed credibility distribution of failure criterion

**Fig. 5:**
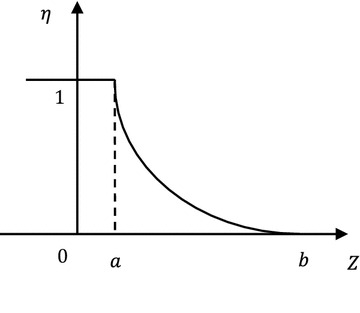
Credibility failure criterion distribution

The symmetric triangular distributed fuzzy failure criterion is turned into lower semi-trapezoid distributed credibility distribution. This distribution converts from possibility distribution interval into a univalent function of credibility measure about limit state. Since credibility under limit state drops significantly with the increase of state function $Z$, this paper employed the following distribution form:

12 $$\eta \left( Z \right) = \left\{ {\begin{array}{*{20}l} 1 \hfill & \quad {{\text{for}} \; Z < a} \hfill \\ {e^{{k\frac{Z - a}{Z - b}}} } \hfill & \quad {{\text{for }}\;a \le Z < b} \hfill \\ 0 \hfill & \quad {{\text{for }}\;Z \ge b} \hfill \\ \end{array} } \right.$$

where coefficients *a*, *b* and *k* could be determined according to requirements on security level of different buildings and corresponding standards based on expert experiences and information entropy method (Shlyakhtenko 2005; Su et al. 2009) (Figs. 3, 4, 5).

### Random fuzzy simulation

In credibility stability failure analysis model of gravity dam, some variables $\left( {H_{1} ,\,H_{2} ,\,\gamma_{c} ,\,\alpha_{2} } \right)$ were vested with a random distribution and others $\left( {f^{{\prime }} ,\,c^{{\prime }} ,\,\alpha_{1} ,\,f_{1}^{'} ,\,c_{1}^{'} ,\,f_{2}^{'} ,\,c_{2}^{'} ,\;\alpha ,\;U_{1} ,\;U_{2} ,\;U_{3} } \right)$ were given with a possibility distribution. If the model contains *k* random variables $\left( {X_{1} ,\;X_{2} , \ldots ,\;X_{k} } \right)$ and $n - k$ fuzzy variables $\left( {X_{k + 1} ,\;X_{k + 2} ,\; \ldots ,\;X_{n} } \right).$

Since all fuzzy input variables are expressed in a fuzzy set, model function was simulated firstly under possibility measure. Then, simulated results were converted into expression of credibility distribution (Baudrit et al. 2005). Considering independent random factors and fuzzy factors in the model, the random fuzzy response of the random fuzzy limit state function of gravity dam is gained through mixed algorithm (Baudrit et al. 2006), which is based on Monte Carlo simulation (Altarejos-García et al. 2012):

1. Generate a random number in $\left[ {0, 1} \right]$ to every random variable. The variable value which takes this random number as the probability distribution is used as one sample $\left( {p^{{x_{1} }} ,p^{{x_{2} }} , \ldots ,p^{{x_{k} }} } \right)_{i} .$
2. Choose one $\alpha$-cut and the membership function of the *j*th sampling is recorded as $\mu_{j}$.
3. Generate maximum and minimum of the response function under this horizontal cut set, $u_{ij}$ and $l_{ij} .$
4. Choose another $\alpha$-cut and repeat Step 2 and Step 3.
5. Choose another random probability distribution sequence and repeat Step 2–4.

**Fig. 6:**
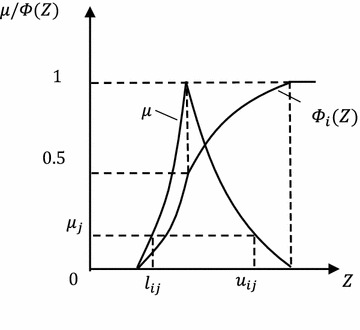
Random fuzzy responses of the *i*th random sample expressed in fuzzy set and credibility measure

For any random sample, the random fuzzy response expressed in membership function is gained through maximum and minimum values of fuzzy response. In other words, for the *i*th $\left( {i = 1,2, \ldots ,N} \right)$ random sample $\left( {p^{{x_{1} }} ,p^{{x_{2} }} , \ldots ,p^{{x_{k} }} } \right)_{i}$ with a membership of $\mu_{j}^{Z} \left( {j = 1,2, \ldots q} \right),$ fuzzy response of state function is expressed as an interval $\left[ {l_{ij} ,u_{ij} } \right].$ Therefore, there are a total of $N \times q$ fuzzy intervals. Based on this random fuzzy response and definition of credibility measure, random fuzzy distribution $\varPhi_{i} \left( Z \right)$ under the *i*th sample expressed in credibility distribution can be gained as(Li and Liu 2009a):

13 $$\varPhi_{i} \left( Z \right) = Cr\left\{ {M < Z} \right\} = \frac{1}{2}\left( {\mathop { \hbox{max} }\limits_{{M_{i} < Z}} \mu \left( {p^{{x_{1} }} ,p^{{x_{2} }} , \ldots ,p^{{x_{k} }} } \right)_{i} + 1 - \mathop {\hbox{max} }\limits_{{M_{i} \ge Z}} \mu \left( {p^{{x_{1} }} ,p^{{x_{2} }} , \ldots ,p^{{x_{k} }} } \right)_{i} } \right)$$

where, $M_{i}$ is arbitrary model function value under the *i*th random sample, $i = 1,2, \ldots ,N$ (Fig. 6).

### Post-processing of simulated results

A series of model credibility distributions under different random samples were gained through mixed algorithm. Based on simulated results, the overall cumulative distribution function (CDF) of the stability failure model is calculated from Eq. (14) (Li and Liu 2009b), which is recorded as $\varPhi \left( Z \right)$:

14 $$\varPhi \left( Z \right) = \mathop {\hbox{max} }\limits_{0 \le \alpha \le 1,y \ge \alpha } \left( {\alpha \wedge \mathop {\hbox{min} }\limits_{Z \in A,} \frac{n\left( Z \right)}{N}} \right)$$

where $n\left( Z \right)$ is random sample size smaller than *Z*, *N* is total random samples, *A* is arbitrary interval of state function value and *y* is credibility value of the interval *A*.

**Fig. 7:**
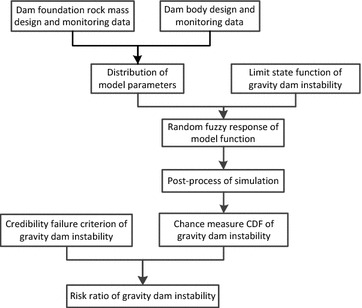
Risk ratio calculation of gravity dam based on credibility theory

The overall instability risk ratio of gravity dam can be known by bringing CDF into Eqs. (2)–(4). When failure criterion is determined and the model function contains both random and fuzzy variables, risk ratio $\tilde{P}$ is calculated from Eq. (2). When the failure criterion is fuzzy and the model function only contain random variables, risk ratio $\tilde{P}$ is calculated from Eq. (3). When the failure criterion is fuzzy and the model function contains both random and fuzzy variables, the overall risk ratio is calculated from Eq. (4). Risk ratios are all determinate real number on an interval $\left[ {0,1} \right]$ (Fig. 7).

## Application

### Basic parameters

**Fig. 8:**
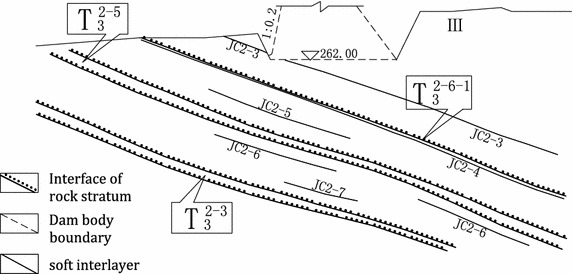
Rock grouping and soft rock strata distribution in the typical foundation section

A serving gravity dam was chosen as the research object. It is a first-grade structure with 100 years of design reference period. The crest elevation, maximum height, normal pool level and level of dead water are 384, 162, 173 and 370 m, respectively. The downstream water level is basically stable. The upstream face above the 295 m elevation is straight and the rest dam body has 1:0.2 slope. The downstream face is vertical and crest width is 12 m. It was constructed on poor rock mass. There’s deep bed rock and the dam foundation has high permeable rate. Two heavy curtains were set on upstream and downstream of the dam. The dam foundation is type-III rock mass. The low-angle weak structural surface (T_3_^2-3^, T_3_^2-5^, JC2-1–JC2-8 etc.) in the dam foundation inclined to the downstream and the low-angle structural surface in downstream resisting foundation inclined to the upstream form the main channel of deep foundation slippage. A non-overflow dam section (elevation: 262 m) on the foundation surface level was chosen in this paper. Rock grouping and soft rock strata distribution in the dam foundation are shown in Fig. 8.

Variables and statistical parameters or fuzzy parameters gained from monitoring data and test data are listed in Tables 1 and 2.

**Table 1:** Basic random variables and fuzzy variables of dam body and foundation

Variables	Variable type	Parameter
Upstream depth $H_{1} \left( {\text{m}} \right)$	Random	$\mu_{{H_{1} }} = 118,\;\sigma_{{H_{1} }} = 2.2$
Downstream depth $H_{2} \left( {\text{m}} \right)$	Random	$\mu_{{H_{1} }} = 8,\;\sigma_{{H_{1} }} = 0.8$
Volume weight of dam concrete $\gamma_{c} \left( {{\text{kN/m}}^{ 3} } \right)$	Random	$\mu_{{f^{{\prime }} }} = 24.0, \;\sigma_{{f^{{\prime }} }} = 0.5$
Shearing friction coefficient of dam foundation surface $f^{{\prime }}$	Fuzzy	$\left[ {0.9,1,1,1} \right]$
Shearing cohesion of dam foundation surface $c^{{\prime }} \left( {\text{MPa}} \right)$	Fuzzy	$\left[ {0.8,0.9,1.0} \right]$
Uplift pressure reduction coefficient of upstream curtain $\alpha_{1}$	Fuzzy	$\left[ {0.16,0.20,0.38} \right]$
Uplift pressure reduction coefficient of downstream curtain $\alpha_{2}$	Random	$\mu_{{\alpha_{2} }} = 0.30,\;\sigma_{{\alpha_{2} }} = 0.03$

**Table 2:** Basic parameters and fuzzy distribution parameters of basement

Stratum	Parameters of AB ($c_{1}$/MPa)		Parameters of BC ($c_{2}$/MPa)		Density γ (kN/m^3^)	α	β
T_3_^2-3^	$f_{1}$	0.35	$f_{2}$	[0.93, 0.98, 1.05]	26	[21°11′, 21°53′, 22°44′]	34°26′
	$c_{1}$	0.1	$c_{2}$	[0.90, 1.00, 1.10]			
T_3_^2-5^	$f_{1}$	0.35	$f_{2}$	[0.93, 0.98, 1.05]	27	[21°35′, 23°26′, 25°21′]	35°15′
	$c_{1}$	0.1	$c_{2}$	[0.90, 1.00, 1.10]			
T_3_^2-6-1^	$f_{1}$	0.35	$f_{2}$	[0.93, 0.98, 1.05]	27	[19°1′, 19°49′, 20°52′]	34°14′
	$c_{1}$	0.1	$c_{2}$	[0.90, 1.00, 1.10]			
JC2-3	$f_{1}$	[0.35, 0.40, 0.44]	$f_{2}$	[0.93, 0.98, 1.05]	26	16°36′	29°5′
	$c_{1}$	[0.10, 0.12, 0.13]	$c_{2}$	[0.90, 1.00, 1.10]			
JC2-4	$f_{1}$	[0.35, 0.40, 0.44]	$f_{2}$	[0.93, 0.98, 1.05]	26	19°18′	33°1′
	$c_{1}$	[0.10, 0.12, 0.13]	$c_{2}$	[0.90, 1.00, 1.10]			
JC2-5	$f_{1}$	[0.35, 0.40, 0.44]	$f_{2}$	[0.93, 0.98, 1.05]	26	21°40′	34°19′
	$c_{1}$	[0.10, 0.12, 0.13]	$c_{2}$	[0.90, 1.00, 1.10]			
JC2-6	$f_{1}$	[0.35, 0.40, 0.44]	$f_{2}$	[0.93, 0.98, 1.05]	27	15°46′	38°51′
	$c_{1}$	[0.10, 0.12, 0.13]	$c_{2}$	[0.90, 1.00, 1.10]			
JC2-7	$f_{1}$	[0.35, 0.40, 0.44]	$f_{2}$	[0.93, 0.98, 1.05]	27	16°2′	36°12′
	$c_{1}$	[0.10, 0.12, 0.13]	$c_{2}$	[0.90, 1.00, 1.10]			

## Result analysis

**Fig. 9:**
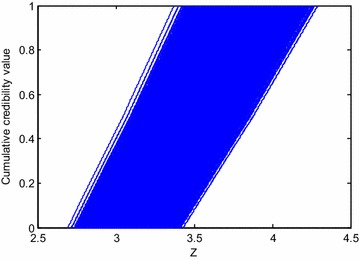
Random fuzzy response to current value of instability state function of dam foundation

**Fig. 10:**
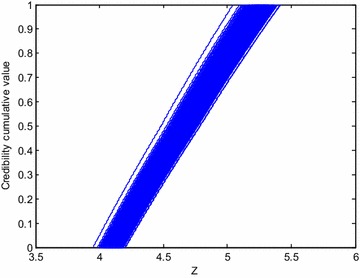
Random fuzzy response to current value of T_3_^2-3^ deep instability state function of the slip surface

Random fuzzy responses of the dam foundation surface instability model and T2-5 deep instability model are shown in Figs. 9 and 10.

Membership function of fuzzy failure state was determined. Considering grade and design specifications of architecture, parameters were determined 2.61, 3.08 and 1.05, respectively. Results are shown in Table 3.

**Table 3:** Credibility instability risk ratios of dam foundation and hypothetical sliding channels

Slip surface	$\tilde{P}$
Dam base	2.25 × 10^−5^
JC2-3	5.64 × 10^−6^
T_3_^2-6-1^	2.68 × 10^−5^
JC2-4	*6.61* *×* *10* ^*−5*^
JC2-5	3.42 × 10^−5^
T_3_^2-5^	5.08 × 10^−6^
JC2-6	0
JC2-7	0
T_3_^2-3^	0

Italic value indicates the risk ratio of the most dangerous sliding surface

**Fig. 11:**
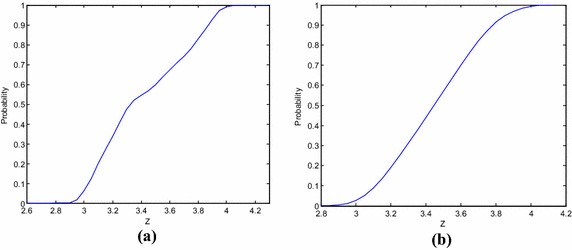
Cumulative distribution of anti-sliding instability probability, **a** JC2-4, **b** dam foundation

CDF of anti-sliding stability state function of dam foundation and deep layers is shown in Fig. 11. It can be seen from calculated results that instability risk ratios of both dam foundation and deep layer are smaller than the acceptable 1 × 10^−4^. Deep T_3_^2-3^, JC2-6 and JC2-7 have zero instability risk ratio. T_3_^2-5^ lies in the fuzzy zone which is about 0.2 m wide and has high fuzzy uncertainty, thus resulting in its high risk ratio although it is deeply buried. The most dangerous sliding surface is on JC2-4, with a risk ratio as high as 6.61 × 10^−5^. This is caused by its low angle and small burial depth as well as the giant upstream sliding block.

**Fig. 12:**
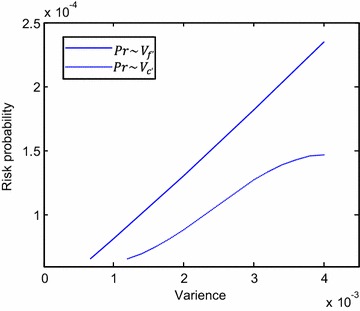
Relationship between risk ratio of dam foundation *Pr* ~ variance of factors $f^{{\prime }}$ and $c^{{\prime }}$

To analyze effect of fuzzy factor *f* and *c* change on risk ratio $Pr,$ sensitivity analysis was implemented by using the following method. Firstly, calculate risk ratio under different variances of *f* while distribution of *c* and mean of *f* are fixed. Draw the curve $Pr\sim\,V_{f} .$ Similarly, draw curve $Pr\sim\,V_{c} .$ Results of sensitivity analysis of stability failure risk assessment of dam foundation are shown in Fig. 12. When variance of fuzzy variable increases, risk ratio of the system increases. This conforms to the general rule.

## Conclusions

Stability failure of gravity dam involves complicated uncertainty. In this article, after adequately considering uncertainty of various factors, a method to analyze stability failure risk of gravity dam and corresponding calculation procedure are proposed. Conclusions are drawn as follows.

To overcome the deficiency of existing risk analysis method that considers randomness and fuzziness separately, this article applies the credibility theory into dam failure and establishes a credibility stability failure risk analysis model of gravity dam to integrate randomness and fuzziness together. Based on Monte Carlo simulation, a mixed algorithm is combined with post-processing of credibility risk analysis mode. And general calculating method is adopted. Stability failure of gravity dam are viewed as hybrid events. Inputs of this calculating method are a series of probability distribution and fuzzy sets. By introducing credibility measure, fuzziness in hybrid variables is mapped from possibility space into the probability space, so risk ratio obtained is represented by a determinate real number.

The example demonstrates that the present method is effective for providing decision support to safety assessment of fuzzy stability of gravity dam. Sensibility analysis results show that credibility risk ratio can sensitively reflect variance change of fuzzy factors. Risk ratio described by credibility measure reflects both randomness and fuzziness and agrees with description habit of traditional probability risk ratio. If there are indexes and standards on credibility risk ratio, credibility theory could become an effective representation tool of fuzzy-random stability failure risk analysis of gravity dam.
